# Molecular Epidemiology of Canine Parvovirus, Europe

**DOI:** 10.3201/eid1308.070505

**Published:** 2007-08

**Authors:** Nicola Decaro, Costantina Desario, Diane D. Addie, Vito Martella, Maria João Vieira, Gabriella Elia, Angelique Zicola, Christopher Davis, Gertrude Thompson, Ethienne Thiry, Uwe Truyen, Canio Buonavoglia

**Affiliations:** * University of Bari, Bari, Italy; † University of Glasgow, Glasgow, Scotland, UK; ‡ University of Porto, Porto, Portugal; § University of Liege, Liege, Belgium; ¶ University of Leipzig, Leipzig, Germany

**Keywords:** Canine parvovirus, variants, MGB probe assays, distribution, Europe, dispatch

## Abstract

Canine parvovirus (CPV), which causes hemorrhagic enteritis in dogs, has 3 antigenic variants: types 2a, 2b, and 2c. Molecular method assessment of the distribution of the CPV variants in Europe showed that the new variant CPV-2c is widespread in Europe and that the viruses are distributed in different countries.

Canine parvovirus type 2 (CPV-2) is a small, nonenveloped DNA virus that emerged suddenly in 1978 as an enteric pathogen of dogs. Two antigenic variants, CPV-2a and CPV-2b, are now distributed worldwide (*1*). A third CPV variant, first named Glu-426 mutant and subsequently renamed CPV-2c, was detected in Italy in 2000 (*2*) and is now circulating in that country together with types 2a and 2b (*3*–*5*). The new variant 2c has also been reported in Vietnam by Nakamura et al., who developed monoclonal antibodies that can identify specifically such a mutant (*6*) and more recently in the United States by Saliki et al. (unpub. data) and in South America (*7*). The antigenic variants differ from the original type CPV-2 for a few amino acids in the VP2 protein, whereas genetic differences among the variants are determined only by residue 426, with types 2a, 2b, and 2c displaying Asn, Asp, and Glu, respectively (*8*,*9*). Recently, minor groove binder (MGB) probe assays have been established for characterization of CPV strains and account for the presence of single nucleotide polymorphisms in the genome of the different variants (*5*). Our objective was to determine the distribution of the CPV variants in different European countries by using the new technology, with particular emphasis on the widespread circulation of CPV-2c in some areas of Europe.

## The Study

A total of 232 fecal samples or CPV isolates were obtained from dogs with diarrhea in Italy (n = 107), Germany (n = 37), the United Kingdom (n = 41), Portugal (n = 31), Belgium (n = 13), Spain (n = 1), Switzerland (n = 1), and the Czech Republic (n = 1). The fecal samples collected in Italy, the United Kingdom, Portugal, and Belgium were CPV-positive according to PCRs performed in local laboratories. Samples were collected during 2005–2006, with the exception of samples from Italy that were collected only in 2006 because other studies had assessed the molecular epidemiology of CPV in the previous decade (*5*). All samples from Germany consisted of cell-culture–adapted CPV strains isolated from dogs with diarrhea in Germany during 1996–2005.

Samples were homogenized (10% w/v) in phosphate-buffered saline (pH 7.2) and subsequently clarified by centrifuging at 1,500× *g* for 15 min. Viral DNA was extracted from the supernatants of fecal homogenates or from the viral suspensions by boiling for 10 min and chilling on ice. To reduce residual inhibitors of DNA polymerase activity to ineffective concentrations, the DNA extracts were diluted 1:10 in distilled water (*10*). CPV DNA titers were calculated by using a real-time PCR, based on TaqMan technology and able to recognize all CPV strains (*10*), whereas characterization of the viral type was obtained by means of MGB probe assays specific for types 2a/2b and 2b/2c (*5*). To rule out the presence of CPV strains of vaccine origin, which are usually type 2 or less frequently type 2b, we subsequently tested samples recognized as types 2/2a and 2b by MGB probe assays that discriminate between vaccine and field strains of CPV (*11*,*12*).

All samples that had CPV-positive results from laboratories located in the countries of sample origin were confirmed by TaqMan assay to contain CPV DNA. CPV infection was also demonstrated in the single samples from Spain, Switzerland, and the Czech Republic (Figure). In Italy, a nearly complete substitution of CPV-2b by CPV-2c was noted; CPV-2a strains were found at low frequency, which confirmed the progressive decrease noted during the past 5 years (*3*–*5*). In contrast, CPV-2b is still prevalent in Germany and Portugal, although CPV-2c is also widespread in these countries. Retrospective analysis of archival samples showed that CPV-2c has been circulating in Germany since 1996. An equivalent distribution of types 2a and 2b was assessed in the United Kingdom, where a single CPV-2c strain was detected. In Portugal, no CPV-2a strain was detected, whereas in Belgium all CPVs were type 2a. The single samples from Switzerland and the Czech Republic were CPV-2a, whereas the sample from Spain was CPV-2c. The original type CPV-2 was detected in 1 sample from the United Kingdom and 4 from Italy, but the latter samples had been collected from dogs shortly after vaccination with a classic type-2–based vaccine (*12*).

**Figure:**
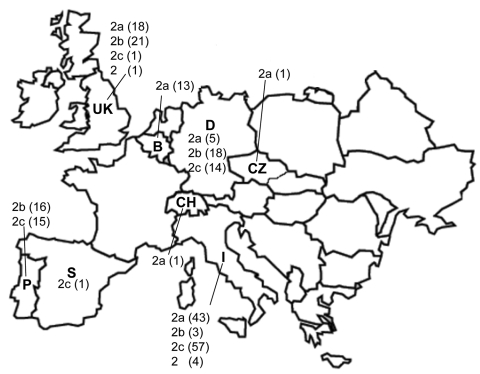
Geographic distribution of antigenic variants of canine parvovirus (CPV) in Europe. CPV-positive fecal samples or viral isolates from different countries were analyzed by molecular methods; strains CPV-2, 2a, 2b, or 2c are indicated for each country by numbers in parentheses. Samples were collected during 2005–2006, except for samples from Italy (2006) and Germany (1996–2005). P, Portugal; S, Spain; UK, United Kingdom; B, Belgium; CH, Switzerland; D, Germany; CZ, Czech Republic.

## Conclusions

Despite its DNA genome, CPV possesses a high genetic substitution rate, similar to that observed for RNA viruses, which is responsible for continuous antigenic evolution and rapid displacement of old types by new antigenic variants (*13*). CPV-2c, which emerged in Italy in 2000 (*2*), is spreading with high efficiency in the dog population of Italy and progressively replacing the antigenic variants 2a and 2b (*3*–*5*). Such a variant has been detected in Vietnam also (*6*), but no information is available on its presence and distribution in other European countries, except for a single case report from Spain (*14*).

Our study shows that the new variant 2c is widespread in some European countries (Italy, Portugal, and Germany) and that it could be detected sporadically in the United Kingdom. In contrast with previous studies of CPV-2a in Europe, our study showed that CPV-2a is most frequent in Belgium, whereas in the United Kingdom, Germany, and Italy, it has been overtaken by CPV-2b or CPV-2c. In Portugal, CPV-2a was not detected at all, but types 2b and 2c were equally distributed. To our knowledge, this is the first report of the new variant 2c in Portugal, the United Kingdom, and Germany. In Germany, CPV-2c was detected in archival samples collected in 1996; thus, it was circulating in Europe 4 years before its first official report in Italy. Such a variable geographic distribution of the CPV variants in Europe may be related to different commercial flows of dogs imported from foreign countries rather than to different vaccination protocols.

The progressive spreading of CPV-2c in the world or, less probably, its independent emergence in different countries, suggests that the Glu-426 mutation provides a certain advantage in viral replication. The replacement of CPV-2 by types 2a and 2b has been associated with increased ability to bind canine transferrin receptors, although it might not rule out the possibility of mutations at residue 426 of the VP2 protein being selected also for their antigenic effects (*1*). Whether the Glu-426 mutation confers benefit in receptor-binding activity is of interest. Another question is whether the CPV vaccines currently used provide full protection against the new variant or whether they should be replaced by homologous vaccines.
